# Cytogenetic and molecular characteristics of rye genome in octoploid triticale (× *Triticosecale* Wittmack)

**DOI:** 10.3897/CompCytogen.v13i4.39576

**Published:** 2019-12-16

**Authors:** Elena V. Evtushenko, Yulia A. Lipikhina, Petr I. Stepochkin, Alexander V. Vershinin

**Affiliations:** 1 Institute of Molecular and Cellular Biology SB RAS, acad. Lavrentiev ave. 8/2, Novosibirsk, 630090, Russia Institute of Molecular and Cellular Biology SB RAS Novosibirsk Russia; 2 Institute of Cytology and Genetics SB RAS, acad. Lavrentiev ave. 10, Novosibirsk, 630090, Russia Institute of Cytology and Genetics SB RAS Novosibirsk Russia; 3 Novosibirsk State University, Pirogova str. 1, Novosibirsk, 630090, Russia Novosibirsk State University Novosibirsk Russia

**Keywords:** Aneuploidy, centromeric histone H3 (CENH3), fluorescence *in situ* hybridization (FISH), remote hybridization, triticale

## Abstract

Alloploidization resulting from remote (interspecific or intergeneric) hybridization is one of the main factors in plant evolution, leading to the formation of new species. Triticale (× *Triticosecale* Wittmack, 1889) is the first artificial species created by crossing wheat (*Triticum* spp.) and rye (*Secale cereale* Linnaeus, 1753) and has a great potential as a grain and forage crop. Remote hybridization is a stress factor that causes a rapid reorganization of the parental genomes in hybrid progeny (“genomic shock”) and is accompanied by abnormalities in the chromosome set of hybrids. The formation of the hybrid genome and its subsequent stabilization are directly related to the normalization of meiosis and the correct chromosome segregation. The aim of this work was to cytogenetically characterize triticale (× *Triticosecale rimpaui* Wittmack, 1899, AABBDDRR) obtained by crossing *Triticum aestivum* Linnaeus, 1753. Triple Dirk D × *Secale cereale* L. Korotkostebel’naya 69 in F_3_–F_6_ generations of hybrids, and to trace the process of genetic stabilization of hybrid genomes. Also, a comparative analysis of the nucleotide sequences of the centromeric histone *CENH3* genes was performed in wheat-rye allopolyploids of various ploidy as well as their parental forms. In the hybrid genomes of octoploid triticale an increased expression of the rye *CENH3* variants was detected. The octoploid triticale plants contain complete chromosome sets of the parental subgenomes maintaining the chromosome balance and meiotic stability. For three generations the percentage of aneuploids in the progeny of such plants has been gradually decreasing, and they maintain a complete set of the paternal rye chromosomes. However, the emergence of hexaploid and new aneuploid plants in F_5_ and F_6_ generations indicates that stabilization of the hybrid genome is not complete yet. This conclusion was confirmed by the analysis of morphological features in hybrid plants: the progeny of one plant having the whole chromosome sets of parental subgenomes showed significant morphological variations in awn length and spike density. Thus, we expect that the results of our karyotyping of octoploid triticales obtained by crossing hexaploid wheat to diploid rye supplemented by comparative analysis of CENH3 sequences will be applicable to targeted breeding of stable octo- and hexaploid hybrids.

## Introduction

Triticale, derived from crossing wheat (*Triticum* spp.) and rye (*Secale cereale* Linnaeus, 1758) was the first synthetic allopolyploid cereal. It incorporates favorable alleles from both progenitor species (wheat and rye), enabling adaptation to environments that are less favorable for wheat yet providing better biomass yield and forage quality (Ayalew et al. 2018). Triticale is a promising model for studying the rapid changes in the hybrid genomes associated with diverse rearrangements in the genomes of parental forms, which occur when parents’ genomes are combined in complex allopollyploid genome (Ma and Gustafson 2008).

The formation of a hybrid genome and its subsequent stabilization are directly related to the normalization of the meiosis process and the correct chromosome segregation (Giacomin et al. 2015). Incompatibility of centromeres of different species appears to be the main reason for the elimination of chromosomes of one of the parental genomes in hybrids (Sanei et al. 2011). According to studies of the last decade, a special role among centromeric proteins is given to the centromeric modification of the histone H3, designated as CENH3 in plants (De Rop et al. 2012, Comai et al. 2017). This is due to the fact that at the molecular level, the most specialized and universal characteristic of the active centromere is the presence of CENH3 instead of the canonical histone H3 in the nucleosomes of centromeric chromatin. As it was shown in some mammalian species and in *Drosophila* Fallén, 1823, in case of its loss the kinetochore does not form and the chromosomes do not segregate correctly during cell division (Talbert et al. 2004).

The variations in the amount and distribution of heterochromatin have facilitated the identification of rye chromosomes in different triticales (Seal and Bennett 1981). The DNA repetitive clone pSc200 has proved to be extremely useful to characterize rye heterochromatin. This highly repeated sequence has been reported to predominantly occupy the subtelomeric regions of all rye chromosome arms. Its monomers of 379–380 bp are organized as long tandem arrays up to hundreds kilobases, and they account for 2.5% of the *S. cereale* genome (Vershinin et al. 1995). Another valuable property contributing to its widespread use in the FISH analysis of triticale karyotypes (Fu et al. 2010, Fu et al. 2013, Fradkin et al. 2013) is the lack of pSc200 hybridization signals on wheat chromosomes.

The aim of this work was to cytogenetically characterize triticale, obtained by crossing *Triticum aestivum* L. line Triple Dirk D × *S. cereale* L., cultivar Korotkostebel’naya 69, by FISH analysis of their rye chromosomes. The parental forms have a number of specific characteristics. The wheat near-isogenic line Triple Dirk D has only one dominant spring allele *Vrn-A1* (Pugsley 1971). The rye cultivar Korotkostebel’naya 69 has the dominant dwarfing gene *Ddw1* (Kobyliansky and Solodukhina 2014). Thus, the triticale lines created using these parental lines are spring short-stemmed plants, which make them a convenient object for studying the triticale karyotypes along consecutive generations. The research was conducted in F_3:6_ generations of hybrids to trace their possible genetic stabilization. Also, we performed a comparative analysis of the nucleotide sequences of the N-terminal tail of the *CENH3* genes in wheat–rye allopolyploids of various ploidy as well as their parental forms.

## Material and methods

### Plant material

Octoploid triticales (genome constitution AABBDDRR) were created by crossing the near isogenic line of common wheat (*Triticum aestivum* L.) Triple Dirk D (genome AABBDD) with diploid rye (*Secale cereale* L.) cultivar Korotkostebel’naya 69 (genome RR) (Stepochkin and Emtseva 2017). Germinating F_1_ seeds were treated with 0.05% colchicine to double the number of chromosomes and obtain allopolyploids. F_2_ generation derived from self-pollination F_1_ plants. F_2_ plants having no less than five grains were selected for further isolated propagation. Generations F_3_ through F_6_ were obtained by self-pollination of plants grown from these seeds. As seeds from each plant were planted separately starting from F_1_, their progeny was designated as lines. Octoploid triticales derived from the Triple Dirk D × Korotkostebel’naya 69 cross were denoted as TDKF3, TDKF4, TDKF5, and TDKF6. All plants were grown under greenhouse conditions at 22 °C/18 °C (day/night) with a 16h day length.

### Cytological techniques and fluorescence in situ hybridization

For chromosome counts in triticale somatic cells, root-tips of seedlings were treated with saturated α-bromonaphthalene solution and visualized through Feulgen staining (Singh 2003). Ten metaphase spreads per each hybrid plant were examined with Axio Star Plus microscope (Carl Zeiss GmbH, Germany). Mitotic chromosome spreads for FISH were prepared as in (Aliyeva et al. 2015). FISH analysis was carried out to identify the chromosome constitution of triticale lines, using pSc200 and pTa71 as probes. Probe pSc200 from rye repetitive sequences was used to determine the rye chromosomes (Vershinin et al. 1995). The rye tandem repetitive sequence pSc200 was labeled with biotin-16-dUTP (Roche Diagnostics, Basel, Switzerland) via PCR (Vershinin et al. 1995). The probe pTa71, containing 45S rDNA (Gerlach and Bedbrook 1979), was labeled with digoxigenin-11-dUTP and nick translational mix (Roche Diagnostics, Basel, Switzerland) according to manufacturer’s recommendations. FISH procedure was performed as described by the Jenkins and Hasterok (2007). Fluorescent signals were visualized using a Zeiss Axio Scope.A1 microscope equipped with filter sets nos. 49, 10 and 20 (Carl Zeiss GmbH, Germany) for detecting DAPI, FITC and Rhodamine signals, 5–10 metaphases per slide were used for the analysis. Images were captured with a Zeiss AxioCam MRm CCD camera and ZEN lite processing package (Carl Zeiss GmbH, Germany). The brightness and contrast of all images were optimized using Adobe Photoshop (Adobe Systems, San Jose, CA, USA).

### RNA extraction and sequence analysis

Total RNA was isolated from leaves of individual young seedlings with TRI Reagent RT (MRC Inc., United States) and treated with RQRNaseFree DNase (Promega Corporation, Madison, WI, USA) according to manufacturers’ recommendations. RNA was reverse-transcribed to cDNA with a RevertAid H Minus First Strand cDNA Synthesis Kit (Thermoscientific). Amplification primers specific to the N-terminal tail of the α*CENH3* gene from rye, wheat (Genbank accession nos. MG384772.1, JF969285.1) and triticale cDNA had been designed in (Evtushenko et al. 2017). The amplification products were cloned by using an InsTAclone PCR Cloning Kit (Thermoscientific). Both strands of 15–20 clones from each parental variety and TDK lines were sequenced using BigDye Terminator Cycle Sequencing chemistry (v. 3.1) on an ABI3100 Genetic Analyzer (Applied Biosystems, CA, USA). Coding sequences of *CENH3* were aligned with online Clustal Omega (Sievers et al. 2011) at http://www.ebi.ac.uk/Tools/msa/clustalo.

## Results

### Cytological and morphological features of octoploid triticale

**Figure 1.**
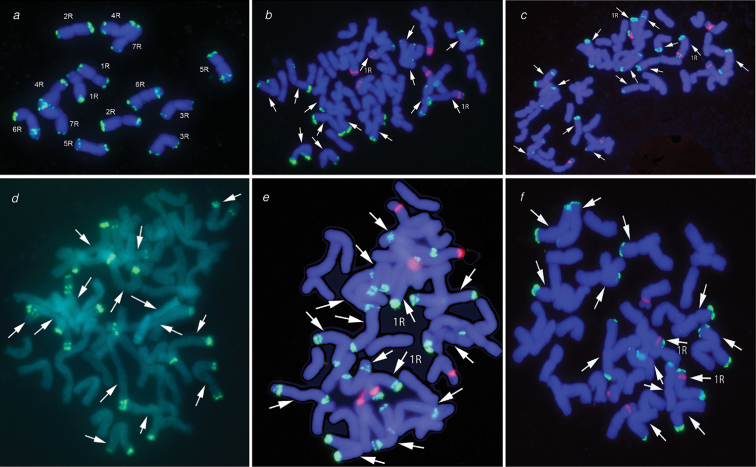
Identification of rye chromosomes by FISH using the pSc200 (green) and pTa71 (red) probes on metaphase chromosomes of the paternal parent and allopolyploid triticale hybrids **a** rye Korotkostebel’naya 69 **b** the line TDK96F3 (56 chromosomes) **c** the line TDK94F3 (55 chromosomes) **d** TDK96.3.F4 (49 chromosomes) **e** TDK96.3.F6 (42 chromosomes) **f** TDK96.3.F6 (41 chromosome). The arrows indicate rye chromosomes.

Karyotype analysis of 30 hybrid plants in the F_3_ generation showed that the number of chromosomes in their somatic cells varies from 53 to 56 (Table 1), with only one plant containing a set of 56 chromosomes. It is interesting that all aneuploid plants, regardless of the number of eliminated chromosomes, contained a complete set of the rye subgenome chromosomes, which was verified by the FISH method using the pSc200 probe (Fig. 1). The pSc200 probe contains a highly repetitive DNA sequence and gives a chromosome-specific FISH pattern allowing the identification of all rye chromosomes (Vershinin et al. 1995). Additionally, the pTa71 probe, which is localized on chromosomes 1R, 1B, 6B, and 5D (Cuadrado et al. 1997), was used to identify the 1R chromosome in allopolyploids. FISH signals of pSc200 probe are localized at both arms of all 14 chromosomes of the parental rye Korotkostebel’naya 69 (Fig. 1a). Plants with a set of 56 and 55 chromosomes were isolated in F_3_ and were designated TDK 96 and TDK 94, respectively.(Fig. 1b, c) The progeny of these plants was analyzed in generations F_4_ (TDK 94, TDK 96), F_5_ and F_6_ (TDK 96). The progeny of the aneuploid plant TDK94F3 (designated as TDK94F4 in Table 1) contained 16.7% of plants with 56 chromosomes. The karyotypes of the remaining plants TDK94F4 were aneuploids with chromosome number varying from 53 to 55, but, as in TDK94F3, they were also represented by complete sets of rye chromosomes (Table 1).

**Table 1.** The chromosome numbers in the karyotypes of somatic cells of triticale lines.

Triticale lines	Generation	Percentages of plants with		Number of chromosomes		
		56 chromosomes	42 chromosomes	Mean	Min-max	Rye
TDK 94	F3	0	0	54.3	53–55	14
TDK 94	F4	16.7	0	54.5	53–56	14
TDK 96	F3	100	0	56.0	56	14
TDK 96	F4	46.2	0	54.5	49–56	14
TDK 96.1	F5	62.5	0	55.3	55–56	14
TDK 96.2	F5	80	0	55.7	55–56	14
TDK 96.3	F5	0	0	44.4	43–47	14
TDK 96.3	F6	0	37.5	42.1	41–45	14

The hybrid plant TDK96F3 containing a set of 56 chromosomes was also reproduced by self-pollination and its progeny (designated as TDK96F4 in Table 1) contained almost three times as many plants with 56 chromosomes (46.2%) as TDK94F4. In F_4_, in most plants of the TDK96 line, the chromosome number varied slightly between 55 and 56 chromosomes, except for two plants that contained 49 chromosomes (Fig. 1d). For analysis of TDK 96 in F_5_, we took two plants with the chromosome number 2n=56 (lines TDK 96.1 and TDK 96.2) and a plant with 49 chromosomes (TDK 96.3) (Table 1). In the lines TDK 96.1 and TDK 96.2 in F_5_, up to 80% of plants maintained a complete set of 56 chromosomes. The progeny of the TDK 96.3 line lost individual chromosomes in the generations F_5_ and F_6_, reaching the minimum number of chromosomes (2n = 41, Fig. 1f) in F_6_, while 37.5% of plants in F_6_ had the chromosome set 2n = 42, representing hexaploid triticale (Fig. 1e). All plants of the TDK96 group, including aneuploid ones, contained complete sets of rye chromosomes; examples of some of them are shown in the Fig. 1b, d–f. However, despite maintaining the complete chromosome sets of the paternal subgenome, the emergence of aneuploid plants and hexaploid triticale in F_5_ and F_6_ generations indicates that stabilization of the hybrid genome in these generations is not complete yet.

**Figure 2.**
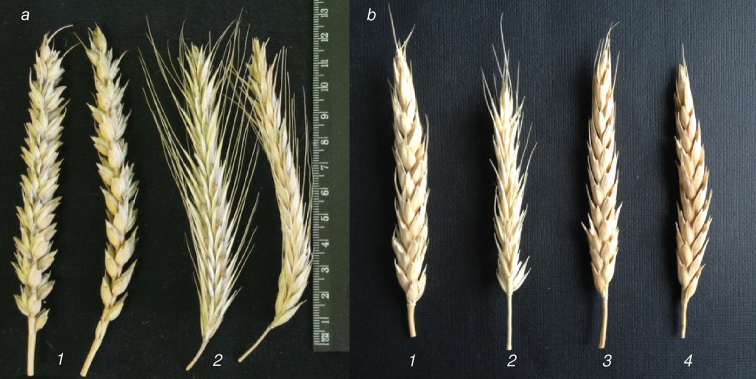
Spike morphology of parental and representative triticale plants with 56 chromosomes **a** parental forms: wheat Triple Dirk D (**1**) rye Korotkostebel’naya 69 (**2**) **b** octoploid triticale hybrids: **1** – TDK 94F4 **2** – TDK 96.1.F5, **3** – TDK 96.1.F5, **4** – TDK 96.2.F5.

This conclusion is confirmed by the analysis of some morphological features in hybrid plants. Figure 2 shows photographs of spikes of parental forms, wheat Triple Dirk D and rye Korotkostebel’naya 69 (Fig. 2a), as well as some hybrid plants with sets of 56 chromosomes (Fig. 2b). The main features that differ between parental forms are the awned spikes and higher spike density characteristic of rye. Some of the hybrid plants (TDK94F4 and TDK96.1.F5) obviously manifest features of rye, while plant TDK96.2.F5 and other plant TDK96.1.F5 manifest features of wheat. Moreover, both TDK96.1.F5 plants are the offspring of one TDK96F4 plant. A variety of morphological features of hybrid plants may be caused by numerous rearrangements leading to the exchange of relatively small regions of the genome between the original parental forms, continuing in each generation.

### Analysis of the structure of centromeric histone CENH3 in octoploid triticale

Intergeneric hybridization in plants is often accompanied by elimination of some chromosomes or whole genomes. One of the putative ways to the emergence of aneuploid plants is disturbances in the functioning of centromeres of one of the parents owing to the differences in the molecular structure of centromeric histones (Sanei et al. 2011). In this regard, we analyzed the molecular structure of the coding sequences of the α*CENH3* N-terminal tail (NTT) in octoploid triticale hybrid plants in F_3_–F_5_ and in the parental forms. Sequencing of randomly selected clones (15–20 clones for each plant studied) of the *CENH3*NTT showed that the nucleotide sequences of wheat and rye are 99% identical, differences were observed only in a few positions. For wheat *CENH3*, these are positions 82 and 84, and for rye – positions 73 and 145. Based on the frequency of specific substitutions in hybrid plants (Table 2), one can assume that predominantly rye CENH3 variants are synthesized in these hybrids. As mentioned above FISH analysis was performed using the pTa71 probe, which confirm the presence of chromosome 1R (Fig. 1b–f) in all hybrid plants, regardless of the chromosome number. Earlier, we have shown that genes encoding the main variants of the rye centromere histone H3 are localized on chromosome 1R (Lipikhina et al. 2017). The rye-specific substitution at the position 73 of the α*CENH3* coding sequence does not occur in the parent wheat variety Triple Dirk D. In triticale plants with different chromosome numbers (56, 49, 43) in generations F_3_-F_5_, the number of α*CENH3* copies carrying this substitution increases from 16 to 57%. The hybrid plants TDK94.2 (54 chromosomes in the karyotype) and TDK92.4 (52 chromosomes in the karyotype) without substitution at the position 73 in the nucleotide sequence of α*CENH3* were sterile. It can be assumed that this substitution affects the formation of the CENH3 structure and is possibly associated with the maintenance of a viable hybrid genome.

**Table 2.** The positions of species-specific non-synonymous SNPs across NTT domain of *CENH3* of wheat, rye and octoploid triticale.

Plants	The percentages of substitutions at positions across NTT domain							
	28	32	73	82	84	99	122	145
*T. aestivum* Triple Dirk D (*AABBDD*), 2n=42		11.1		55.6	55.6	27.8		
*S. cereale* Korotkostebel’naya 69 (*RR*), 2n=14			8.7	7.4	7.4	30.4		21.7
**Octoploid triticale F_3_**								
Plant 1, (2n=56), TDK 96	5.6		16.7	5.6	5.6	11.1	5.6	5.6
Plant 2 (2n=52), TDK 92.4	16.7		–			16.7	33.3	
Plant 3 (2n=54), TDK 94.2	30	10	–	10				
**Octoploid triticale F_4_ (derived from F_3_, plant 1)**								
Plant 1 (2n=56), TDK 96.1	5		35	10	10	10	15	5
Plant 2 (2n=56), TDK 96.2	10		30	10	10		10	
Plant 3 (2n=49), TDK 96.3		6.7	33.3	6.7	6.7	6.7		6.7
**Octoploid triticale F_5_ (derived from F_4_, plant 1)**								
Plant 1 (2n=56), TDK 96.1.1		7.1	50				7.1	
**Octoploid triticale F_5_ (derived from F_4_, plant 3)**								
Plant 2 (2n=43), TDK 96.3.1			57.1	7.1			14.3	7.1

## Discussion

Various chromosomal rearrangements in allopolyploid hybrids are among the most frequently described effects of remote hybridization. Significant genomic changes at remote hybridization cause instability of the hybrid genome and chromosome number imbalance. An imbalance of the hybrid genome results in a high percentage of aneuploid plants immediately after the crossing (Kalinka and Achrem 2018). In octoploid triticale, which is cytologically highly unstable, the proportion of aneuploids reaches 83% (Lukaszewski and Gustafson 1987). In our case, almost all F_3_ plants, except one, were aneuploids with different numbers of eliminated chromosomes (Table 1).

Deletions and translocations of individual chromosomal regions and chromosome arms are also among the most common chromosomal alterations and have been found in the cytogenetic analysis of wheat-rye substitution and addition lines (Alkhimova et al. 1999, Fu et al. 2013), triticale, and their progeny from crosses triticale × wheat (Appels et al. 1982, Ma and Gustafson 2006). In addition to these rearrangements, cases of formation of minichromosomes and chromosomes with multiple centromeres have been described (Fu et al. 2013). Because of the meiotic instability previous studies have shown that hexaploid lines could be spontaneously derived from primary octoploid triticales, with the retention of most of A-, B- and R-genome chromosomes and the elimination of most of the D-genome chromosomes and even chromosomes of the whole wheat D-genome (Dou et al. 2006, Li et al. 2015). These results suggest that the stability of the D-genome is more strongly affected by the R genome in the octoploid triticale, comparing to the A and B genomes of common wheat. In our experiments, spontaneous emergence of plants with 42 chromosomes was also observed. These plants were found in the progeny of F_5_ aneuploid plants that lost several chromosomes. The high percentage of aneuploids and the preferred elimination of wheat D-genome chromosomes in the first generations after remote hybridization should be attributed to stable signs of triticale.

Data on the effect of “genomic shock” on the chromosomes of the rye subgenome in hybrid plants are contradictory. Early studies indicated that both rye and wheat chromosomes contributed to aneuploidy (Merker 1971), as opposed to the results reported by Müntzing (1957) and Stutz (1962), who concluded that meiotic disorders and aneuploidy of triticale mainly involved rye chromosomes. Weimarck (1974) pointed out that the proportion of eliminated rye and wheat chromosomes was about 1:3. Since the chromosome ratio in the rye and wheat genome is also 1:3, it indicates that chromosomes are eliminated in equal proportions. In recent works an opposite trend has been reported. For example, in F_5_ and F_8_ generations of octoploid triticale higher elimination extent of wheat chromosomes than that of rye has been found, taking into account the genomic proportions, since the ratio was on average 1:11 (Kalinka and Achrem 2018). Up to 62% of the plants had a complete set of rye chromosomes, while only about 4% had a complete set of wheat chromosomes. Among rye chromosomes, 4R and 5R were preferably eliminated (Kalinka and Achrem 2018). In advanced lines of octoploid triticale, about 70% of plants had 2n=56, however, almost all plants lost the 2R chromosome and the short arm of the 5R chromosome. The reduction of rye chromosomes was compensated by an extra pair of 2D (or 2A) chromosomes in plants with 2n=56 (Cheng and Murata 2002). The appearance in our experiments of hybrid octoploid plants of triticale with different proportions of aneuploid plants retaining complete sets of rye chromosomes indicates a complex, difficult to predict nature of inheritance of parental subgenome chromosome balance. There is a tendency indicating that F_3_ plants containing complete chromosome sets of the parent subgenomes more readily maintain the chromosome balance, and more easily adapt the rye subgenome chromosomes to heterogeneous wheat cytoplasm. In subsequent generations, the percentage of aneuploidy in the progeny of such plants gradually decreases. Thus, the total chromosome number in combination with the complete set of paternal chromosomes can serve as a diagnostic indicator of the prospects for further breeding of such plants.

Differences in the CENH3 structure between the parental forms allow us to judge the regulation of the expression level of the parental protein forms in a new genomic environment that arises in a hybrid cell in case of remote hybridization. The first study of the possible relationship between differences in the CENH3 structure in parental forms and the processes of parental genome chromosome segregation during the division of hybrid cells was carried out on hybrids obtained by crossing cultured barley *H. vulgare* L. and its closest wild relative *H. bulbosum* Linnaeus, 1756. The CENH3 molecules were not included in the centromeres of *H. bulbosum* chromosomes, which were herewith inactivated and eliminated from hybrid embryos. Perhaps this was due to significant differences in protein structure between the barley species, especially in the structure of the N-terminal tail (Sanei et al. 2011). Unlike barley species, the coding sequences of α*CENH3*s in various rye and wheat species have a very high (95–99%) identity (Evtushenko et al., 2017), which noticeably complicates the search for interspecific differences and, accordingly, determining the nature of inheritance of the differences in hybrid genomes.

Our results on *CENH3* sequences analysis in hybrid plants (Table 2) indicate that both parental genomes are involved in the formation of the structure of this molecule in octoploid triticale. In the generations F_3_-F_5_, the proportion of plants with polymorphism at the position 73 in hybrid plants increases (from 16 to 57%), which is typical only for *CENH3* clones obtained from the rye genome. Of great importance for the discussion of these data is an extensive analysis of the expression of rye genes in cDNA isolated from various tissues of hybrid allohexaploid triticale plants obtained by crossing *T. turgidum* × *S. cereale* (Khalil et al. 2015). The classes of missing (or silent) rye genes have been identified in diploid rye and triticale. A comparison of diploid rye and hexaploid triticale revealed 112 rye cDNA contigs (~ 0.5% of the total amount), which were not determined by expression analysis in any of the triticale tissues, although their expression was relatively high in diploid rye tissues (Khalil et al. 2015). It is important to note that the rye genes not expressed in triticale had significantly less homology to the corresponding homeologs in the genomes of wheat or other *Triticum* species than 200 randomly selected rye genes. Thus, rye genes with a low similarity to their homeologs in *Triticum* genomes are more likely to be repressed or absent due to deletions in the allopolyploid genomes. This conclusion is in good agreement with our results. High identity of rye and wheat CENH3 sequences does not inhibit the expression of both parental forms in the hybrid genomes of octoploid triticale.
